# Case Report: Multifocal EBV-Associated Diffuse Large B-Cell Lymphoma in a Patient With 6-MP Associated Lymphopenia With TPMT Deficiency

**DOI:** 10.3389/fped.2022.881612

**Published:** 2022-05-06

**Authors:** Lara Müller-Scholden, Frank Deinlein, Matthias Eyrich, Paul Gerhardt Schlegel, Verena Wiegering, Matthias Wölfl

**Affiliations:** Pediatric Oncology, Hematology and Stem Cell Transplantation Program, University Children's Hospital Würzburg, Würzburg, Germany

**Keywords:** TPMT deficiency, EBV, lymphoma, PTLD, maintenance therapy

## Abstract

**Introduction:**

EBV associated lymphoproliferative disorders (EBV LPD) are a known complication following solid organ or hematopoietic stem cell transplantation. The disturbance of the immune system leads to a lack of control of latent EBV-infected B-cells, as control by T-cells is mandatory to prevent uninhibited cell proliferation. EBV LPD in other settings is less frequent and etiology and pathogenesis are not completely understood.

**Case Presentation:**

We present the case of an 18-year old adolescent suffering from lymphoblastic T-cell lymphoma who developed a life-threatening EBV associated B-cell lymphoma while he was under therapy with 6-MP (6- mercaptopurine). An underlying homozygous TPMT (thiopurine S-methyltransferase) deficiency with subsequent insufficient degradation of 6-MP was identified as contributory for the development of a distinct lymphopenia leading to EBV LPD. The patient was successfully treated by discontinuation of 6-MP and initiating rituximab monotherapy.

**Discussion:**

Rare cases of EBV LPD during therapy with 6-MP are reported in patients with leukemia, but no data about TPMT pharmacogenomics are available. In contrast the disease development in the presented case may be explained by the iatrogenic immunosuppression in the context of TPMT deficiency. While using 6-MP testing of genetic variations is not required for every protocol, although the use of thiopurines in patients with TPMT deficiency can cause severe immunosuppression. Our case suggests that insufficient degradation of 6-MP can have significant consequences despite dose reduction.

**Conclusion:**

When using thiopurines, TPMT genetics should be initiated and strict drug monitoring and dose adjusting must be performed by a specialized center.

## Introduction

EBV associated lymphoproliferative disorders (EBV LPD) comprise of a spectrum ranging from polyclonal to monoclonal diseases, potentially culminating in aggressive B-cell lymphoma (1). EBV-associated post-transplant lymphoproliferative disease (PTLD) is a severe complication following solid organ (SOT) or hematopoietic stem cell transplantation (HSCT). In rare cases, EBV associated lymphoma in other settings is associated with primary or secondary immunodeficiency when T-cells fail to control proliferation of EBV-infected B-cells (2). There are some reports of children suffering from leukemia who developed an EBV LPD during maintenance therapy, but etiology and pathogenesis are not completely understood (3–6).

We present the case of an 18-year old adolescent with homozygous TPMT-deficiency (thiopurine S-methyltransferase) suffering from lymphoblastic T-cell lymphoma who developed a life-threatening multifocal EBV associated B-cell lymphoma while under therapy with dose-adjusted 6-MP (6- mercaptopurine). He was successfully treated by discontinuation of 6-MP and initiating rituximab monotherapy.

## Case Description

At the age of 17 years, the patient was diagnosed with a lymphoblastic T-cell lymphoma (stage III), manifesting as mediastinal mass and mesenteric lymph nodes. Until the diagnosis, the patient had always been healthy. There were no indications of an increased susceptibility to infections. He was treated with poly-chemotherapy according to treatment guidelines NHL BFM 2012. Serology of EBV and CMV was consistent with prior infection. The tumor masses rapidly responded to chemotherapy, but prolonged myelosuppression prompted testing of TPMT pharmacogenomics which revealed a homozygous polymorphism (^*^3A/^*^3C). As a consequence, maintenance therapy per protocol with oral 6-MP was reduced to 10% of the maximum 6-MP dose (50 mg/m^2^) and regularly adjusted according to blood cell counts. MRI, 1 year after diagnosis, showed complete remission. At his own request, the patient then emigrated abroad while still taking oral maintenance chemotherapy (6-MP and MTX). The last documented blood cell counts were low, but within the target range defined by the study protocol (leukocytes, granulocytes, lymphocytes, thrombocytes).

Five months later, he returned in reduced general condition, presenting with diffuse abdominal pain and massive weight loss (30 kg in ~5 weeks, equivalent to 25% of his body weight). Two weeks prior to his return, he had been admitted to a general hospital abroad for suspected pneumonia with symptoms of coughing and high fever. The chest x-ray showed multiple round pulmonary lesions. He reported having regularly taken the prescribed medication in the recommended dose (6-MP, methotrexate, cotrimoxazole for PCJ prophylaxis).

His markers of inflammation were elevated (CRP 116 mg/l, ESR 98 mm/h), LDH was increased (520 U/l) and pancytopenia with distinct lymphopenia (leucocytes 2.900/μl, Hb 9.1 g/dl, thrombocytes 90.000/μl, granulocytes 2.450/μl, lymphocytes 116/μl) was noted. Flow cytometry (fluorescence-activated cell sorting) did not show a relevant B-/T- or NK-cell gap. The immunoglobulin levels were within normal range [IgG 763 mg/dl (reference range 700–1,550 mg/dl)]. Low-level reactivation of EBV (EBV-DNA PCR 610 copies/ml) and CMV (CMV-DNA-PCR positive, <300 copies/ml) was detected. Work-up for other infections (including HIV, TBC, fungi) was negative. Bone marrow biopsy showed a representative bone marrow cytology without blasts. Except for the described lymphopenia, there was no further evidence of an underlying immunodeficiency. Imaging of chest, abdomen and pelvis confirmed the presence of multiple round lung foci and revealed an extensive abdominal tumor with suspected ileal perforation. Explorative laparotomy was performed, which revealed tumor masses within the ileum, infiltrating the bladder wall. En-bloc resection of the ileal segment including the bladder wall and termino-terminal ileo-ileostomy was executed. Somewhat unexpectedly, pathology revealed an EBV-associated, diffuse large B cell lymphoma, strongly expressing CD20, CD79a, CD30, and co-expression of CD45. EBV early RNA (EBER) *in situ* hybridization was markedly positive and the proliferation activity (Ki- 67) was above 70%.

**Figure 1:**
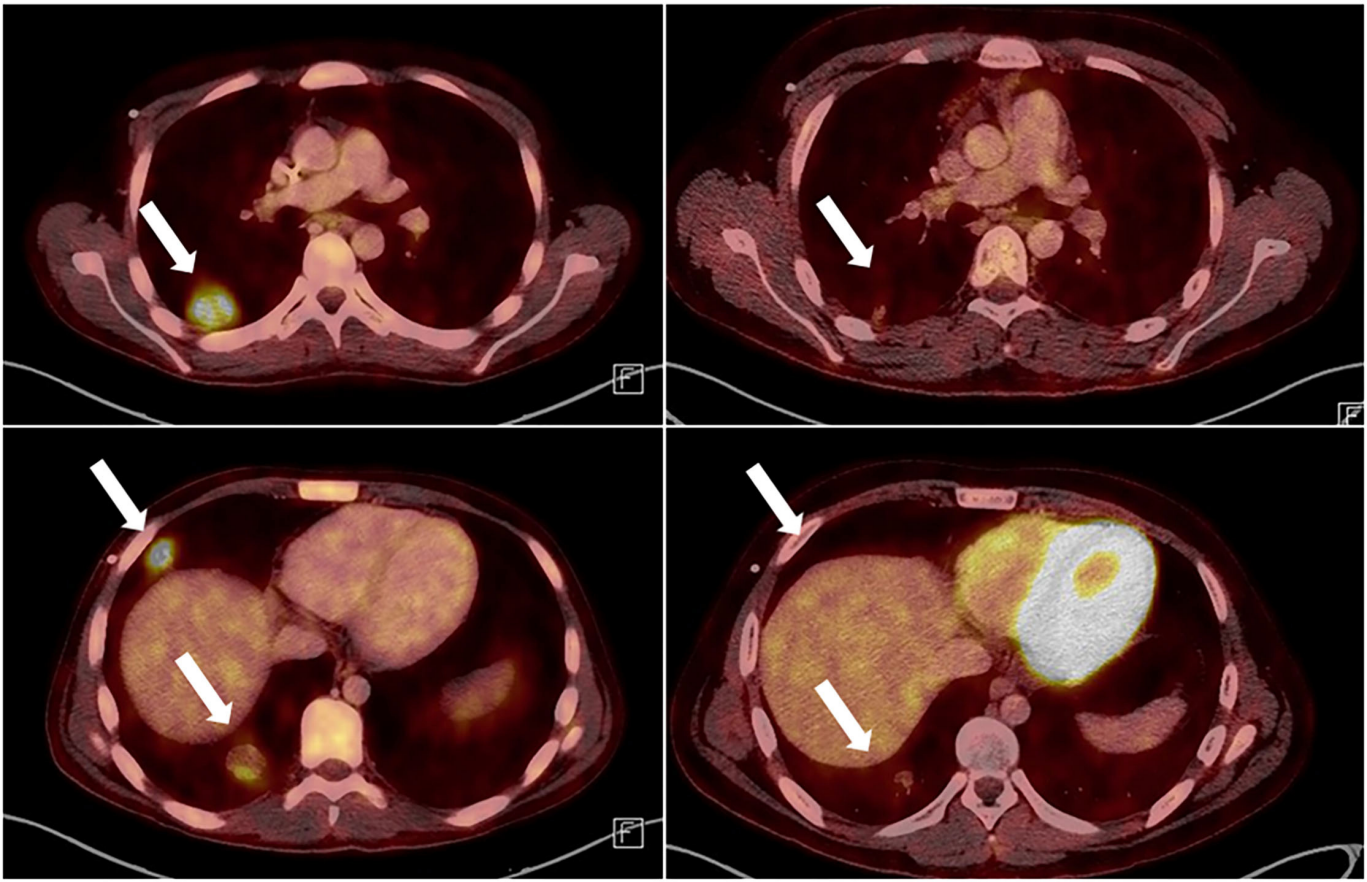
Thoracic FDG-PET CT prior to treatment **(left side)** and after 5 cycles or Rituximab **(right side)**. Lesions marked with arrows. The luminous structure in the lower right picture is the left ventricle, which shows an increased metabolism compared to the right picture, because it is contracting. This FDG-PET accumulation has nothing to do with the metabolism of the tumor cells.

Immediately at the time of emergency admission, 6-MP maintenance therapy had been stopped, leading to a prompt increase of lymphocyte counts within 3 weeks. 17 days after abdominal surgery, with histology confirmed, 5 weekly cycles of rituximab were administered (each with 375 mg/m^2^). After two cycles, a significant regression of the pulmonary lesions was documented via MRI, and EBV viral load as well as CMV virus load in the peripheral blood became negative and remained negative thereafter. After completion of therapy, FDG-PET indicated complete metabolic response without evidence of vital lesions (Figure 1). After discharge from inpatient care, the patient received close follow-up through our oncology outpatient clinic. FDG-PET examinations were performed every 2 months and EBV-PCR was measured monthly for a total period of 6 months. Twelve months post diagnosis, the patient is in excellent clinical condition without additional therapy.

## Methods

We performed a retrospective chart review and a collection of the patient's medical records including laboratory parameters, imaging, histopathological findings and clinical outcome. Written informed consent was obtained from the individual for the publication of any potentially identifiable images or data included in this article. The case was put in context to the existing published literature to this topic.

## Discussion

EBV-LPD is a known complication in patients with congenital or acquired immunodeficiency, but especially after SOT or HSCT. In this case, distinct lymphopenia with subsequent insufficient control of latent EBV-infected B cells was contributory for the development of multifocal EBV LPD. This lymphopenia was considered to be caused by insufficient degradation of 6-MP as a consequence of the underlying homozygous TPMT deficiency.

Some protocols, especially for childhood ALL include routine TPMT pharmacogenomics prior to the start of the therapy, whereas for other protocols testing is not mandatory. As a result, implications of an altered thioguanine metabolism is mostly studied in ALL patients (6). Thiopurines are antimetabolites that lead to cell apoptosis by integrating a false nucleotide into the DNA. The metabolites are inactivated by the enzyme TPMT (6). Variants in the TPMT gene lead to enzyme deficiency, which impairs the inactivation of thiopurines. Particular in individuals with a homozygous variant, accumulation of thioguanine nucleotides in hematopoietic tissue can lead to myelosuppression with potentially fatal outcome (6, 7).

**Figure 2:**
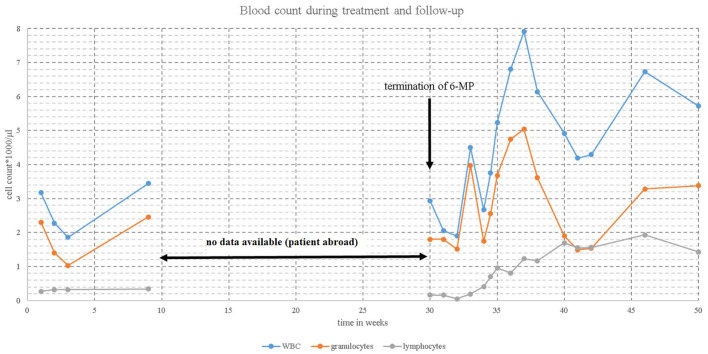
Blood count [WBC 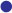 (white blood cell), 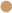 granulocytes, 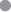 lymphocytes] during maintenance therapy and after termination of mercaptopurine (6-MP).

In post-transplant, EBV-associated lymphoproliferative disease, the first step in therapy generally is the reduction of immunosuppression—if possible. As a second step, rituximab monotherapy often is successful, but sometimes additional chemotherapy may be needed. As a last resort, third party EBV-specific cytotoxic T lymphocytes may be used (2, 8–10). The key element in our patient was to immediately stop 6-MP therapy, thereby lifting immunosuppression demonstrated by increasing lymphocyte counts (Figure 2). Monotherapy with rituximab in our patient led to complete remission.

LPD in non-transplant patients is rare and could be linked to different primary or acquired immunodeficiencies. In a survey conducted in Great Britain from 1994 to 2004, a total of 98 LPD cases were analyzed, of which ~1 quarter had occurred without association to transplantation. These were largely due to congenital immune disorders. Patients with ALL or B-NHL on maintenance therapy accounted for only a very small proportion (about 3% of all cases each) (11).

A few case reports describe the occurrence of EBV LPD during therapy with 6-MP in patients with leukemia, but do not include data on TPMT pharmacogenomics (3–5, 12, 13). The authors assume that patients with ALL under maintenance therapy are susceptible to EBV LPD because of an induced—not further specified—T cell defect (4, 5). Our case is associated with the iatrogenic immunosuppression in the context of TPMT deficiency.

The TPMT variant ^*^3A/^*^3C is one of the most common genetic variants with homozygous non-functional alleles (7). For this patient, a dose reduction of 6-MP was recommended and blood cell counts were regularly checked. Drug level monitoring might have helped to monitor the patient even better. However, one pitfall in this case was the exclusive lymphopenia in the patient: lymphocyte counts ranged between 300 and 500/ul, whereas WBC, granulocytes and platelets were within accepted limits. Lymphocytes do not have a salvage pathway for purine synthesis, but in severe cases one would expect that TPM toxicity may likewise affect neutrophils (14, 15). Therefore, apart from the explicit dose reduction to 10% due to the TPMT polymorphism, there was no further reduction (nor any reason for it) during his therapy in Germany. At what point this balance failed during the subsequent 5 months abroad remains unclear. When he returned as an emergency patient, he needed a blood transfusion upon admission to stabilize his condition in the first place, which unfortunately prevented drug level monitoring, as TMP metabolites are measured in erythrocytes. We were not able to completely resolve, why 6-MP had not been reduced earlier during the time abroad. One pitfall may have been, that 6-MP more frequently leads to neutropenia rather than isolated lymphopenia, as mentioned above. Also absolute leucocyte numbers, had been normal, and only the differential blood count revealed the underlying problem.

This remarkable case, in which immunosuppression, possibly induced by maintenance chemotherapy, resulted in life-threatening consequences, illustrates the importance of TPMT pharmacogenomics when using thiopurines. Appropriate dose reduction and careful drug monitoring are essential. Moreover, it highlights the importance of specialized centers, familiar with the difficulties of maintenance therapy for early detection and treatment of potential complications.

## Data Availability Statement

The original contributions presented in the study are included in the article/supplementary material, further inquiries can be directed to the corresponding author/s.

## Ethics Statement

Written informed consent was obtained from the individual(s) for the publication of any potentially identifiable images or data included in this article.

## Author Contributions

LM-S and MW formulated the conclusions and wrote the report. FD, ME, PS, and VW assisted in gathering data and editing the manuscript. All authors contributed to the article and approved the submitted version.

## Funding

This work was supported by a research grant from the Tour of Hope Foundation.

## Conflict of Interest

The authors declare that the research was conducted in the absence of any commercial or financial relationships that could be construed as a potential conflict of interest.

## Publisher's Note

All claims expressed in this article are solely those of the authors and do not necessarily represent those of their affiliated organizations, or those of the publisher, the editors and the reviewers. Any product that may be evaluated in this article, or claim that may be made by its manufacturer, is not guaranteed or endorsed by the publisher.
